# The Teeth and Oral Cavity of Pregnant Women

**Published:** 1890-03

**Authors:** John S. Marshall

**Affiliations:** Chicago


### THE DENTAL REGISTER.

VOL. XLIV.] MARCH, 1890. [No. 3.

### COMMUNICATIONS.

### The Teeth and Oral Cavity of Pregnant Women.

BY JOHN S. MARSHALL, M.D., CHICAGO.

Read in the Section of Oral an l Dental Surgery, at the Fortieth Annual Meeting of the American Medical Association, June, 1889.

From time almost immemorial, pregnancy has been recognized as having a deleterious effect upon the teeth; and this opinion has become so general that it has been crystallized into the familiar adage—"for every child a tooth." All writers upon obstetrics and the care of pregnant women, recognize the fact that during the period of gestation and lactation women are more liable to suffer from diseases of the teeth and neuralgia of the facial region than at any other time; but the causes, prevention, or mitigation ot these diseases has received but little attention; in fact, it seems to be considered by most of them that they are among the inevitable consequences of gestation and that little can be done to relieve them.

With this view I do not fully agree, for I believe *much can* be done to prevent or relieve the suffering incident to caries and the loss of the teeth, and the neuralgic conditions of the face and mouth so common at this period. But in order to appreciate the causes and the treatment of these disorders, we must have in mind the changed conditions of the organism. The pregnant state is characterized by many and varied changes in the general system, and in special organs, the most marked of which are those of the uterus, the genitals and mammary glands, but which it is not necessary for us to take time to describe, as they are generally well understood. The changes which bear the closest relationship to our subject, are those of nutrition affecting the condition of the blood, the bones, the teeth and the excretions and secretions.

Pregnancy usually exercises a favorable influence upon nutrition; after the third or fourth month the appetite improves, adipose tissue is laid on and the body weight is progressively increased, so that at the completion of gestation it is about one-thirteenth greater than before.[1] Many times, however, the reverse of this is met with; nutrition is so disturbed as to cause considerable emaciation and general debility.

The *blood* is increased in volume during the second half of gestation, as was demonstrated by Spiegelberg and Gescheidlen[2] in experiments upon pregnant bitches. Authorities, however, are not agreed as to changes in the character of the blood. Andral,[3] Nasse,[4] Meyer,[5] and others claim that the watery elements and the white corpuscles are increased and the red corpuscles diminished. Ingerslev[6] could detect no diminution in the number of the red corpuscles; while on the other hand Fehling[7] found the hæmoglobin and the red corpuscles increased. Hypertrophy of the left ventricle, of temporary character, sometimes occurs as the result of increased labor thrown upon the heart consequent upon the augmentation of the blood mass. This was first made known by Larcher[8] in 1828. Venous congestion, varices, and swelling of the lower extremities and arterial hyperæmia of the upper half of the body is a frequent accompaniment of pregnancy. The causes of these conditions are not fully understood, but were attributed by De Cristoforis[9] to pressure of the gravid uterus upon the iliac veins preventing the return of blood to the inferior vena cava, and pressure upon the descending aorta, thus obstructing the normal flow of blood to the lower extremities and causing arterial hyperæmia of the upper portion of the body.

---

1 Hirst's American System of Obstetrics, p. 353.
2 Ibid., p. 345.
3 Animals de Chymie et de Physique, Juellet, 1842.
4 Archives of Gyn. Bd. x, p. 338.
5 " Untersuchungen uber die Zeianderungen des Blutes in der Schroangerschaft."
6 Centralb. f. Gyn., 1879, p. 635.
7 Archives of Gyn., Bd. xxviii, Heft, 3, p. 454.
8 Hirst's Amer. Syst. of Obstetrics, p. 346.
9 Ibid.

Changes in the constituent elements of the *bones* are of common occurrence as the result of malnutrition. Dalton[10] says: "Next to the chloride of sodium, the phosphate of calcium is considered the most important mineral ingredient of the body. It is met with universally in every tissue and every fluid"—and "whenever the nutrition of the bone during life is interfered with from any pathological cause, so that its phosphate of calcium becomes deficient in amount, a softening of the osseous tissue is the consequence, by which the bone yields to external presssure and becomes more or less distorted." Softening or decalcification of the bones undoubtedly takes place in a limited extent in many cases of pregnancy. In fractures occurring during gestation, union is often delayed, sometimes until after delivery. Padieu[11] describes a case in which fractures of the tibia and fibula occurred nine days after the suppression of the menses, and in which union was delayed until the end of gestation. The process of union began ten days after delivery and was completed at the end of a month. On the other hand, the bones of the extremities frequently increase in length, and osteophytes are sometimes formed, both intra- and extra-cranial.

According to Forster,[12] in a little more than one-half of the cases of pregnancy examined after death, there were found deposited upon the inner surface of the skull, thin lamellæ of a bone-like formation, composed of phosphate and carbonate of calcium, and measuring from one-sixth to one-half of a line in thickness. These formations are most commonly found upon the frontal and parietal bones, in the neighborhood of the sulcus falciformis and the arteria meningia media, and usually occur after the third month. These formations are not, however, exclusively an accompaniment of pregnancy, for they are frequently found associated with tuberculosis, and might therefore be classed as effects of insufficient nutrition, rather than over-nutrition.

The *pelvis*, though looked upon at all other times as a comparatively solid framework, frequently becomes relaxed in its

10 Dalton's Physiology, fifth edition, p. 58.
11 Journal de Medicine et de Chirurgie pratique, 1887.
12 Handbuch der patholog. Anat., Vol. ii, p. 945.

articulations during pregnancy so that the sacro-iliac and pubic joints become movable. Luschka[13] demonstrated that these articulations were true joints. Lenoir[14] found in twenty-two pregnant women between the ages of eighteen and thirty-five, movement of the sacro-iliac and pubic articulations. Occasionally such movement is so great as to make locomotion difficult or impossible.

Lawson Tait[15] is authority for the statement that the *thyroid gland* frequently increases in size during pregnancy, and that he noted in districts where goitre was endemic, and in women in whom there existed the predisposition, pregnancy may produce a temporary form of the disease or furnish the exciting cause for the development of the permanent disease.

The *spleen*, in all probability, is increased in size during gestation.[16] The functions of the *kidneys* are also heightened and in consequence of this they become somewhat larger.[17] The urine undergoes change both in quantity and quality; the aqueous elements are increased in consequence of the augmentation in the blood mass and the high arterial tension. Winckel found no change in the amount of urea, sodium chloride, sulphates and phosphates daily excretcd. Chalvet and Barlemont, on the other hand, found the chlorides increased in quantity and the phosphates, sulphates, urea, uric acid, creatin and creatinin diminished in amount. Lehmann and Donne[18] have suggested that the cause of this deficiency was due to the extra demand for these substances in the development of the fœtus. Glucose and albumen are not infrequently present in the urine during pregnancy. An increase in the *salivary* secretions is often a noticeable symptom. *Ptyalism*, when present, manifests itself early and usually disappears spontaneously between the third and fourth months. It occasionally persists, however, in an aggravated form during the entire period of gestation and even for several weeks after, and the amount secreted may be so great

13 Die Anatomie des Menschlicken, Bunkens, Tubingen, 1864.
14 Hirst's American System of Obstetr cs, p. 341.
15 Obst. Jour. of Great Brit in and Ireland, 1875.
16 Wood's Handbook of Medical Sciences, Vol. vi, p. 5.
17 Hirst's American System of Obstetrics, Vol. i. p. 334.
18 Ibid., Vol. i, p. 354.

as to endanger the life of the patient. The qualitative changes in the saliva during pregnancy are sometimes quite marked. The water is increased, while the organic and inorganic elements are diminished. Schramm reported one case in which the ptyalin was entirely absent. In those cases in which excessive ptyalism is manifested the buccal mucous membrane is more or less inflamed, the parotid, submaxillary and sublingual glands are swollen and tender, and quite painful when their functions are especially excited. Fetor is not present, and the absence of this symptom distinguishes it from mercurial ptyalism. The cause of this disorder is in all probability a reflex neurosis, and it frequently reappears in successive pregnancies. *Gingivitis* is another common accompaniment of pregnancy, and is often present when there is no indication of salivation. Pinard is of the opinion that it is more common among multiparæ than primiparæ. This opinion is based upon seventy-five cases, of whom forty-three were multiparæ and thirty-two primiparæ. In the former the disorder was present thirty-one times, in the latter fourteen times. Gingivitis is characterized by redness and tumefaction of the gums, and a tendency to bleed on slight pressure or friction, as in mastication or the use of the tooth-brush; while the secretions of the mucous gland are often decidedly acid in reaction, as may be easily proven by litmus paper. Treatment should consist of saline cathartics and astringent and antacid mouthwashes, and thorough cleanliness of the mouth and teeth

*Phagedenic pericementitis* is occasionally present during pregnancy, and right here let me call your attention to the fact that this disease is often associated with rheumatic affections, diabetes mellitus and albuminuria, but just how it is associated in relation to cause and effect with kidney affections I am unable to say, but would suggest that it is due to the accumulation of effete products in the system, possibly of uric acid, urea, and the like. This disorder is characterized by tumefaction of the gums, which have a purplish tint along the margins, the festoons are swollen and detached from the teeth, and bleed on the least provocation; many of the teeth are loose in their alveoli, from which pus constantly exudes, and in a majority of the cases examination of the roots of these teeth will show them to be more or less covered with calculus, the character of which is still in dispute, some claiming it is salivary, others serumal. After a little time the gums begin to recede from the necks of the teeth, and it is not by any means rare for this disease to progress so rapidly that one or more teeth are lost by the disorder before the completion of gestation. The disease usually persists after delivery, but not with such rapid progress as before. Treatment consists in removing the deposits from the affected teeth, incising the gums down to the bottom of each pocket to give drainage for the escape of the discharges, the application to the parts of a saturated solution of iodine in wood creosote, and the use of astringent and antiseptic mouth washes.

The changes in the *nervous system* due to the effects of pregnancy are many and varied, the character being determined largely by the individual susceptibility of the patient to nervous irritation. As a rule, the emotions are more easily excited than at other times. Some are bright and happy, others despondent, moody or peevish. Functional disorders of the special senses are not infrequent, vision may be impaired, the hearing affected, or perversions of taste and smell may occur. Neuralgic affections are quite common during pregnancy, and most frequently affect the face and head. Odontalgia, tic douleureux, local anæsthesia and paresis are the most common forms.

*Odontalgia* is generally the result of dental caries which penetrates to the pulp and sets up an acute and chronic pulpitis, or may be caused (as recently stated by Dr. W. W. Allport[19]), by hyperæmia of the pulp, due to the augmented volume of blood, increased arterial pressure and capillary hyperæmia of the upper half of the body. In proof of this statement by Dr. Allport, I would call attention to the fact that a brisk cathartic will often relieve, or entirely control an attack of odontalgia or a congestive headache, by simply relieving general arterial tension and local hyperæmia of the parts by depleting the circulation;

19 Paper on "Neuralgia," read before the American Medical Association, June, 1889.

this relieves the blood pressure upon the nerve filaments of the pulp or the meninges of the brain, and the pain ceases. Tic douloureux, local anæsthesia and paresis of the facial region are generally considered to be due to a reflex neurosis, but the point made by Dr. Allport is worthy of careful consideration. Odontalgia due to pulpitis can be controlled by devitalizing the pulp, and the tooth be made permanently useful by appropriate after-treatment; a temporary stopping is all that should be attempted until after delivery if the attack occur during the latter months of gestation, but in the earlier months there need be no fear, in a majority of cases, in performing a permanent operation if it be not too fatiguing.

Nervous disorders due to the changed condition of the system during pregnancy usually disappear soon after delivery, but they occasionally persist during lactation. The treatment of neuralgic conditions of reflex origin, and nervous disorders in general, should be relegated to the family physician, as they need general treatment and therefore come more especially within his province. Quinine, iron, or other general tonic treatment, however, is indicated, with change of climatic surroundings.

The statement has been made that softening and decalcification of the bones takes place to a limited extent in certain cases of pregnancy, but whether the change is due to a failure of the nutritive processes to supply the waste constantly going on in all the tissues of the body, or an actual resorption of the inorganic materials to supply these elements to the forming fœtus, I am unable to state, but am of the opinion that it is the result of both faulty nutrition and the lack of supply of the proper elements to the system. In some cases it is certainly the direct result of faulty nutrition incident to the pregnant state. In others inability to take food, as in those cases where for months the stomach is in such an irritable condition as to make it impossible to retain sufficient food to properly nourish the body; while in others it is the result of improper diet.

Many women are in the habit of discarding from their aliment during pregnancy all those foods which contain an abundance of calcium salts, and restrict themselves, as nearly as possible, to a fruit diet, believing that by such practice the bones of the child will be perfectly calcified, and thus parturition will be robbed of much of its suffering. There is no scientific evidence that such a result is obtained; while on the other hand, as in those cases affected with hyperemesis, though the child when born may be small and much emaciated, it has the appearance of being properly formed and its bones as dense as in the majority of normal pregnancies, and the fact seems to be pretty well established that the osseous framework of the fœtus is formed in such cases at the expense of the maternal organism.

We have no knowledge that a chemical analysis has ever been made upon the teeth of the same individual before and during pregnancy in cases like those just described; and without such an analysis it could not be dogmatically asserted that there was or was not a quantitative change in the inorganic materials of the teeth, but reasoning from analogy, we would expect to find the same conditions existing in the teeth that are undoubtedly present in the bones, and brought about by the same causes.

Clinical observation would seem to substantiate such a proposition, for every observing and thoughtful dentist, of even limited experience, could relate cases in which a material and an appreciable softening of the dentine occurred during the pregnant state of many of his patients. This condition is an important predisposing cause of caries, and is often present in adults and in school children of both sexes, as a result of overwork, anxiety or excessive mental strain.[20]

The principal exciting cause of caries during pregnancy, as in all other cases, is the ever-present lactic acid fermentation, but its action is greatly augmented by the changed conditions of the salivary and buccal secretions. The acids found in the secretions of the mouth as a result of the disorders incident to the pregnant state, are acetic, hydrochloric, uric and oxalic. Consequently in those cases where the dentine has been rendered abnormally soft—deficient in calcium salts—there is likely to be a rapid breaking down of tooth structure during prengancy and lactation.

Long, tedious operations, like the restoration of form in

20 Jacob L. Williams, Journal Am. Med. Assoc., 1885.

decayed teeth in gold, are inadmissible during gestation. All operations upon the teeth at such times should be as free from pain and fatigue as possible, from the fact that in certain cases miscarriage might be the result. Caries of the teeth can be arrested by the use of temporary fillings like Hill's Stopping, or oxy-phosphate cement, until such time as the patient is in condition to bear the nervous strain incident to large gold operations. Among the most valuable of preventive measures are a thorough and frequent use of the tooth-brush and floss silk at least three times per diem, supplemented by tooth powders and antacid mouth washes.

I am aware that I have not discussed this subject as its great importance would seem to deserve, and had time permitted I should have been glad to have elaborated many points that have been only just alluded to. I trust, however, that some of these points may receive more extended treatment in the discussion that shall follow.

